# Overpotential for CO_2_ electroreduction lowered on strained penta-twinned Cu nanowires†

**DOI:** 10.1039/c5sc02667a

**Published:** 2015-08-19

**Authors:** Zhengzheng Chen, Xu Zhang, Gang Lu

**Affiliations:** a Department of Physics and Astronomy, California State University Northridge CA 91330 USA ganglu@csun.edu

## Abstract

Based on first-principles calculations, we predict that penta-twinned Cu nanowires (NWs) are superior to conventional Cu catalysts for CO_2_ electroreduction. The penta-twinned NWs possess a combination of ultrahigh mechanical strength, large surface-to-volume ratios and an abundance of undercoordinated adsorption sites, all desirable for CO_2_ electroreduction. In particular, we show that the penta-twinned Cu NWs can withstand elastic strains orders of magnitude higher than their conventional counterpart, and as a result their CO_2_ electroreduction activities can be significantly enhanced by elastic tensile strains. With a moderate tensile strain, the bias potential for methane production at a decent current density (2 mA cm^−2^) can be reduced by 50%. On the other hand, the competing hydrogen evolution reaction can be suppressed by the tensile strains. The presence of H at the NW surface is found to have a minor effect on CO_2_ electroreduction. Finally, we propose to use graphene as a substrate to stretch deposited Cu NWs.

## Abstract 2

Penta-twinned Cu nanowires have a strain-tunable low overpotential for CO_2_ electroreduction.

## Graphical abstract

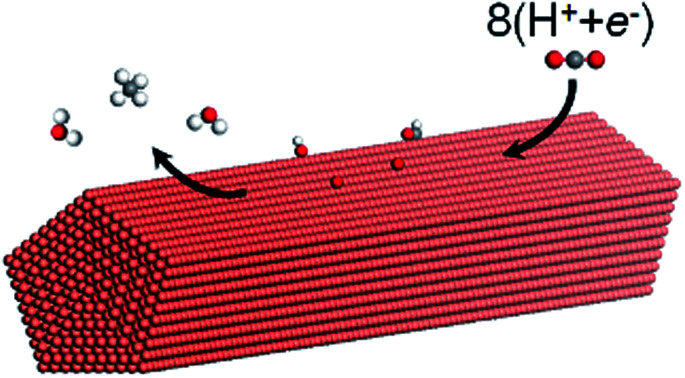

## Introduction

(Photo)electroreduction of CO_2_ is a critical and attractive technology that could positively impact the global carbon balance by recycling CO_2_ into renewable fuels and useful chemicals.^1–4^ As CO_2_ is an extremely stable molecule, its reduction demands efficient catalysts and sufficient energy input. In particular, CO_2_ electroreduction involves multiple proton-coupled electron processes that are kinetically sluggish, in need of high overpotentials.^5,6^ However, no catalyst is presently known that can reduce CO_2_ to hydrocarbons with sufficient efficiency, selectivity and stability. In fact, copper and its alloys are the only metals that have been shown to be capable of producing significant quantities of hydrocarbons from CO_2_, but they do so inefficiently with high overpotentials.^7–10^ For example, a potential of −0.8 V was required for the onset of CH_4_ production from CO_2_ in experiments.^11^

Significant research effort has been devoted to the electroreduction of CO_2_ at metal electrodes.^12–24^ On the experimental side, much focus has been placed on nanostructures, which have been demonstrated to exhibit superior catalytic activities to their bulk counterparts. For instance, Reske *et al.* reported enhanced CO_2_ electroreduction activities on Cu nanoparticles with decreasing nanoparticle sizes.^25^ Elevated electroreduction activities have also been observed on Ag, Pd, Pt–Pd nanoparticles and Au nanowires, to name just a few.^26–30^ On the theoretical side, Peterson *et al.* have carried out a pioneering study to understand the CO_2_ reduction mechanism on a Cu electrode and to elucidate the origin of high overpotentials on Cu.^15^ More specifically, they used a well-established computational hydrogen electrode (CHE) model^31^ in conjunction with first-principles calculations to determine free energy diagrams of relevant reaction paths in transforming CO_2_ to CH_4_. From the free energy diagrams, they concluded that the protonation of CO to form CHO was the rate-limiting step in the electroreduction of CO_2_ to CH_4_, and the corresponding overpotential was estimated as −0.74 V, agreeing very well with the experimental value of −0.8 V.^11^ These and other previous studies have inspired and indeed form the basis of the present study.

**Fig. 1:**
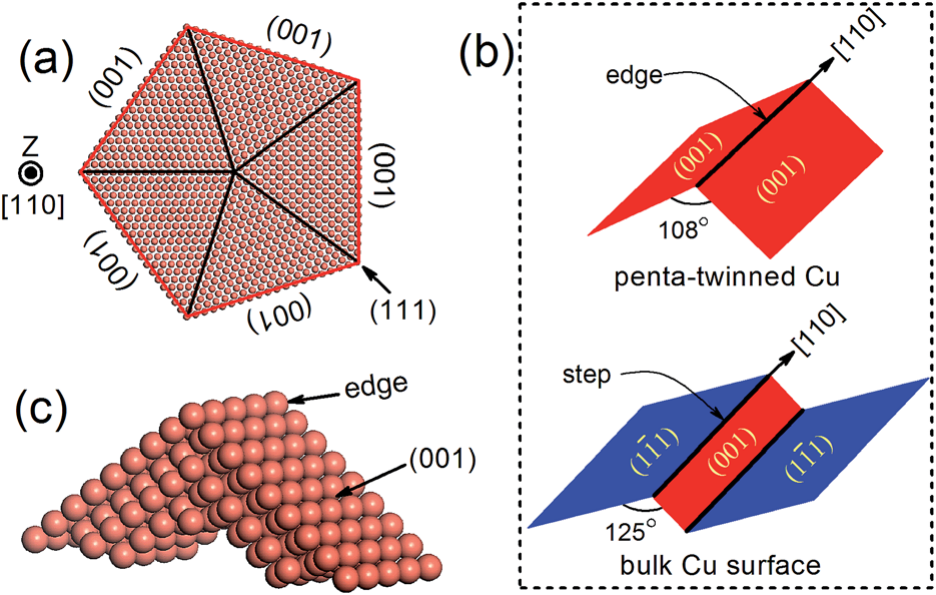
(a) Atomic structure of a penta-twinned Cu NW with *d* = 8 nm. (b) Comparison between the NW edge and the (111) surface step. (c) The supercell used in DFT calculations.

Recently, penta-twinned Cu nanowires (NWs) have been synthesized in aqueous solutions with glucose as the reducing agent and hexadecylamine serving as the capping agent. These Cu NWs can be produced in a relatively large quantity and with high purity and good uniformity.^32,33^ More interestingly, the NWs have a penta-twinned atomic structure, bound by ten {111} facets on the two ends and five {100} facets with a pentagonal cross-section (see Fig. 1 for schematic). The average diameter of the NWs is about 24 ± 4 nm, and their length can vary in a range from several tens to hundreds of microns. Herein, we predict that the penta-twinned Cu NWs may be used to catalyze CO_2_ electroreduction with substantially lower overpotentials and higher mechanical stability than the conventional Cu electrode. Our work was motivated by three considerations: (1) the nano-twinned materials are known to exhibit ultrahigh mechanical strengths compared to their coarse-grained counterparts,^34,35^ therefore the Cu NWs are expected to be mechanically more stable than bulk Cu. In particular, the NWs could withstand much higher elastic strains, which makes it feasible to tune their catalytic activities with the elastic strains.^36–40^ (2) The Cu NWs have much larger surface-to-mass ratios than their coarse-grained counterpart. (3) It is known that low-coordinated adsorption sites at {111} 〈110〉 steps are the most active for CO_2_ reduction in Cu,^41,42^ and these steps form a so-called vicinal surface of the {211}-type. All five facets and edges of the penta-twinned Cu NWs are less close-packed than the {111} surfaces, and thus are expected to be active for CO_2_ electroreduction.

## Method

The atomic structure of a penta-twinned Cu NW is displayed in Fig. 1(a). We have used the circumscribed circle diameter (*d*) of the Cu NW to represent the size of the NW. Five twin boundaries of (111) type and five surfaces of (001) type are indicated by black and red lines, respectively. The [110] edge of the NW flanked by the two intersecting (001) facets is displayed in Fig. 1(c). The [110] (111) step predicted as the most active site on the Cu surface for CO_2_ electroreduction^15^ is shown schematically in the lower panel of Fig. 1(b). It is useful to contrast the atomic structures between the surface step and the edge of the NW. Both are in the [110] direction and on a (001) plane. The main differences between them are: (1) the dihedral angle of the NW is 108°, less than that of the surface step (125°); (2) the surface on the other side of the step is of {111} type instead of (001), as in the NW. The computational model used in the DFT calculations is shown in Fig. 1(c).

The DFT calculations are performed using the Vienna *ab initio* simulation package.^43–45^ The revised Perdew–Burke–Ernzerhof (RPBE) exchange-correlation functional^46^ and the projector-augmented wave pseudopotential^47^ are used in the calculations. Brillouin-zone integration is performed with a 2 × 6 × 1 *k*-mesh according to the Monkhorst–Pack scheme.^48^ The energy cutoff is 400 eV and a Fermi–Dirac smearing width of 0.02 eV is employed. The optimized atomic geometries are achieved when the forces on all atoms are smaller than 0.03 eV Å^−1^. The computational unit cell consists of 150 atoms shown in Fig. 1(c). It contains one [001] edge, two (001) facets and six atomic layers in the [001] direction. Our DFT calculations follow closely the work of Peterson *et al.* on the conversion of CO_2_ to CH_4_. The free energy corrections, including zero-point energy and entropy, are included in our calculations. The solvation contribution to the free energy is taken from the work of Peterson *et al.*^15^ The Computational Hydrogen Electrode (CHE) model is used in the present work. The CHE model^31^ is a technique to determine the free energy change at each reaction step that involves proton–electron transfer as a function of applied electrical potential. This technique allows the study of electrochemical reactions without the explicit treatment of solvated protons, and has been widely used in the literature.^49–51^ More computational details can be found in the ESI.† The free energy difference Δ*G*(*U*) between two adjacent intermediates is expressed as a linear function of the applied potential *U vs.* the reversible hydrogen electrode (RHE):^8,15^

1 Δ*G*(*U*) = Δ*G*(*U* = 0) + *neU*,

where *n* is the number of proton–electron pairs transferred to CO_2_ and *e* is the positive elementary charge. The rate-limiting step is the one with the maximum Δ*G*(*U*), and the overpotential, *U*_op_, is thus defined as2
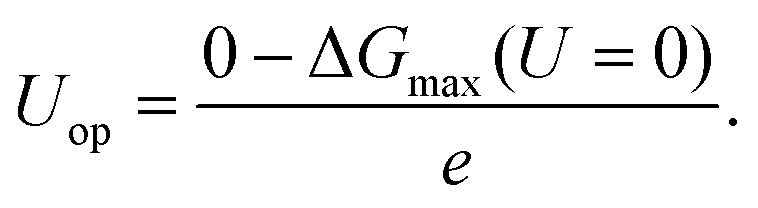


For convenience, in the following we will take the absolute value of *U*_op_ as the overpotential. We have carefully examined the numerical convergence with respect to the size of the computational unit cell. Two larger unit cells with 204 and 250 atoms have been used to compute *U*_op_, and the results are within 8 meV of that of the 150-atom supercell.

## Results and discussion

Various hydrocarbons can be produced on a Cu electrode, including methane, ethylene, formic acid, methanol, *etc.*^11–13,42^ Among them, methane is the dominant product at high bias potentials.^11^ Hence in this paper, we focus on methane and ethylene, with more emphasis on methane.

### Production of methane

**Fig. 2:**
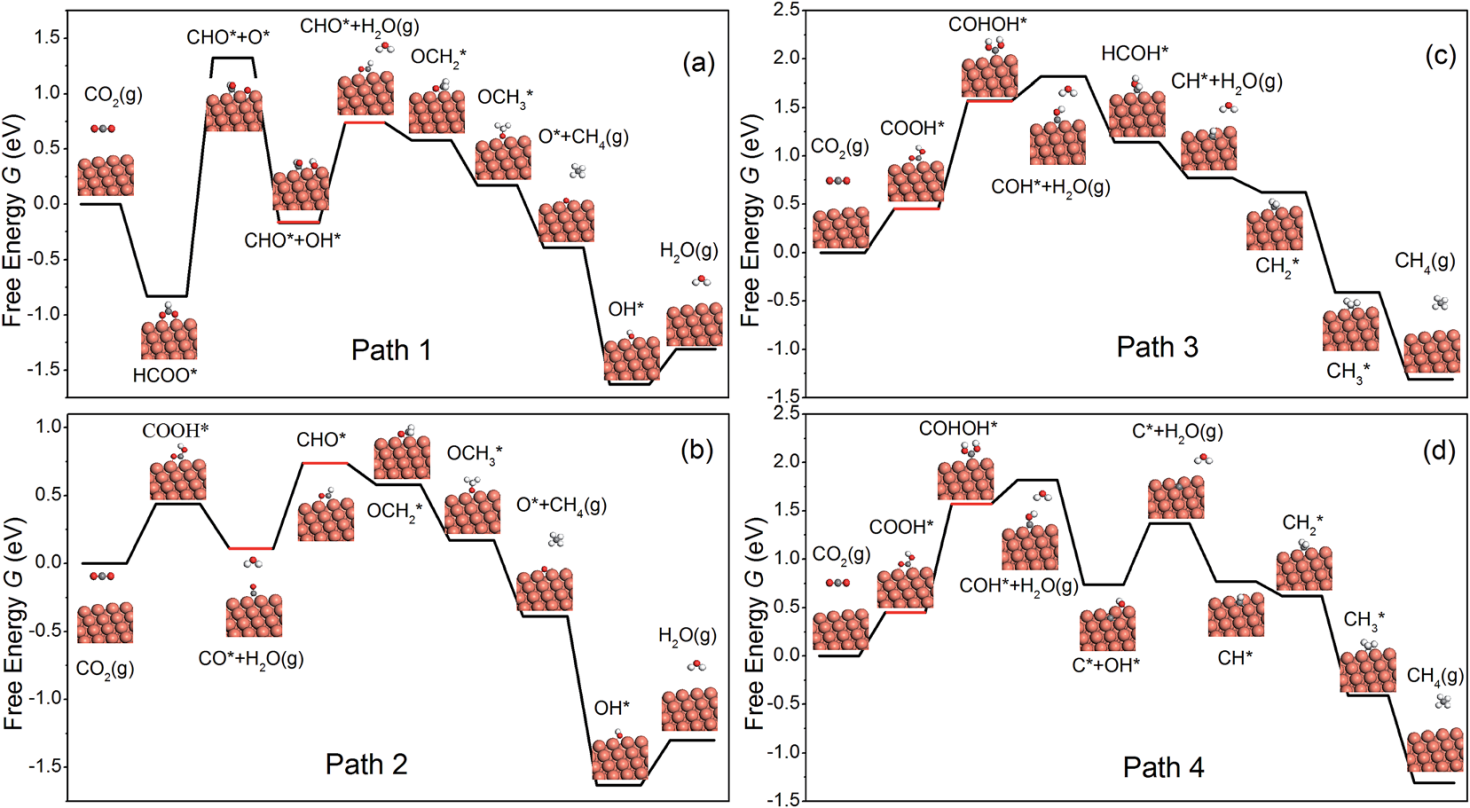
The free energy diagrams for the formation of CH_4_ on the NW edge.

Similar to Peterson's work,^15^ various intermediates in CH_4_ production on the NW edge have been considered in this work, and four reaction pathways are identified as the most relevant. The free energy diagram of each pathway has been calculated and is shown in Fig. 2. The reaction intermediates along each pathway are labeled in the diagram along with schematics of the reaction species. Particular attention should be paid to the overpotential-determining step, indicated by the red horizontal lines. This is the rate-limiting step involving coupled proton–electron transfer with the highest free energy barrier along a given pathway. The overpotential is found to be 0.91, 0.63, 1.22 and 1.22 V, for Paths 1–4, respectively. Among them, Path 2 has the lowest overpotential for CH_4_ production, and the overpotential-determining step is the protonation of CO, *i.e.*, CO* → CHO*. Between Paths 1 and 2, the main difference is the formation of the first intermediate, HCOO on Path 1 and COOH on Path 2. The formation of HCOO requires a dissociation step, HCOO* → CHO* + O*, which is energetically very expensive (2.15 eV) and renders the reaction unlikely to happen. Path 3 and Path 4 have the same overpotential, determined by the protonation of COOH*, *i.e.*, COOH* → COHOH*. As shown in Fig. 2(c and d), the protonation causes OH to detach from the surface and form a monodentate COHOH, which is also energetically costly and requires a higher overpotential. Since Path 2 has the lowest overpotential among all the pathways for CH_4_ production, we will focus on it in the remainder of the paper. It is worth noting that Path 2 on the NW edge is identical to the lowest overpotential pathway on bulk Cu as reported by Peterson *et al.*^15^

**Fig. 3:**
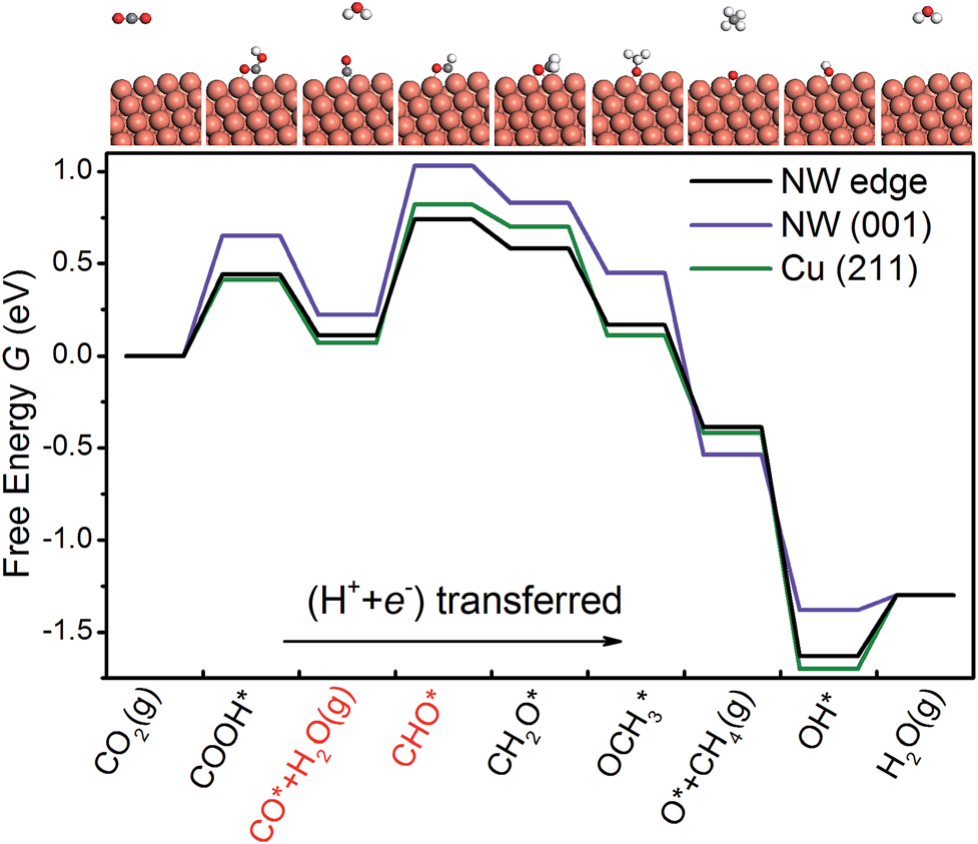
The free energy diagrams of CO_2_ electroreduction on the NW edge, the NW (001) facet and the Cu (211) surface at 0 V *vs.* RHE. The two intermediates shown in red indicate the overpotential-determining step. The large orange spheres represent Cu atoms, and the small spheres represent O (red), C (gray) and H (white) atoms.

As shown in Fig. 3, we are able to reproduce the main results of Peterson *et al.* on the bulk (211) surface. In particular, the overpotential obtained from our calculations (0.75 V) matches very well with that of Peterson *et al.* (0.74 V). We have also calculated the free energy diagram on the NW (001) facet along the same pathway. We find that the free energy diagrams on the NW are similar to that on the (211) surface, and in each of the three cases, *U*_op_ is determined by the protonation of the adsorbed CO. Crucially, we find that the overpotential on the NW edge is 16% lower than that on the bulk (211) surface. This reduction is due to much stronger binding of CHO on the NW. Furthermore, the overpotential on the NW (001) facet is predicted as 0.81 V, 8% higher than that on the (211) surface. However, as shown later, by applying tensile strains to the NWs, the overpotential on the NW (001) facet could be reduced substantially below that on the (211) surface.

### Production of ethylene

We have also calculated the free energy diagrams for the production of ethylene on the NW edge, and the results are detailed in the ESI.† Two reaction pathways have been examined and the overpotential for C_2_H_4_ synthesis is determined by the dimerization of CH_2_, *i.e.*, CH_2_* + CH_2_* → C_2_H_4_*. The overpotential is estimated as 0.82 V, 30% higher than that for CH_4_ production.

### Hydrogen evolution reaction

**Fig. 4:**
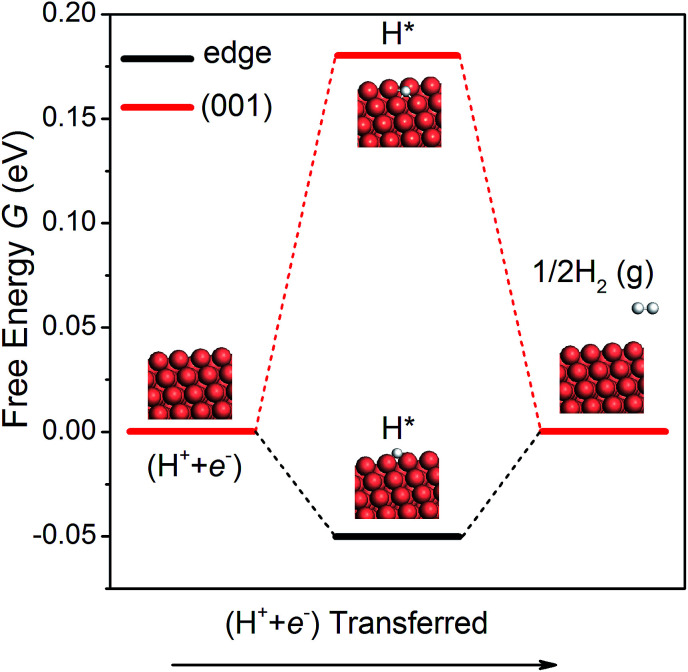
The free energy diagrams of the HER on the NW edge and (001) facet.

The hydrogen evolution reaction (HER) is a competing side reaction that consumes H ions and hinders the CO_2_ electroreduction. As the HER is known to be a more kinetically facile reaction,^18^ it is necessary to examine it on the NWs. Following the work of Nørskov *et al.*,^52^ we have calculated the free energy diagrams for the HER on the edge and the (001) facet of the NW, as shown in Fig. 4. Since there is only one intermediate (H*) in the pathway, the overpotential is simple to compute. On the edge and the (001) facet, the overpotential is found as 0.05 and 0.18 V, respectively. Note that the overpotential-determining step at the edge and at the (001) facet is different – desorption of H_2_ on the edge *vs.* adsorption of a proton–electron pair on the facet. Since the overpotential of the HER is lower than that of CH_4_ production, H_2_ will be formed first on the Cu NWs under a small bias potential. However, as pointed out by others,^15,16^ intermediates O* and OH* in CO_2_ electroreduction compete with H* in the HER to occupy the adsorption sites. Since the adsorption of O and OH is much stronger than that of H on the edge of the NWs, the HER could be effectively suppressed. Moreover, a recent theoretical study suggested that CO generated in CO_2_ reduction could also block the adsorption sites for the HER.^53^ Therefore, CO_2_ electroreduction on the Cu NWs is expected to be active despite the competing HER.

### Presence of H on NWs

**Fig. 5:**
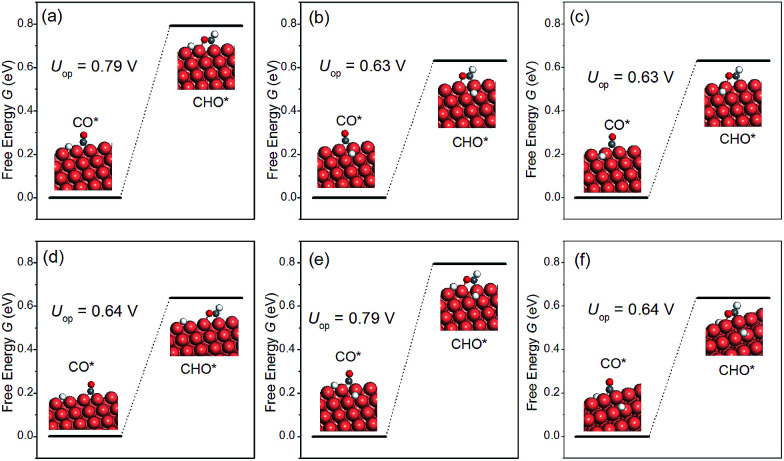
The overpotential-determining step as a function of H configuration on the NW. (a) A single H atom on the edge and the 1st NN to the adsorbed CO; (b) a single H atom on the facet and the 1st NN to CO; (c) a single H atom on the facet and the 2nd NN to CO; (d) a single H atom on the edge and the 2nd NN to CO; (e) one H on the facet and another H on the edge; both are 1st NNs to CO; (f) two H atoms on the facet and the 1st NNs to CO.

The discussion thus far has been confined to the clean surface. However, the surface of NWs is likely to be contaminated by H species. For example, Mistry *et al.* reported that H coverage could affect CO_2_ electroreduction on Au nanoparticles.^54^ Here we examine the presence of H atoms on the overpotential of CH_4_ production. More specifically, six H configurations in the overpotential-determining step are considered, including: (i) a single H atom on the edge as the first nearest neighbour (NN) to the adsorbed CO; (ii) a single H atom on the facet as the first NN to the adsorbed CO; (iii) a single H atom on the facet as the second NN to the adsorbed CO; (iv) a single H atom on the edge as the second NN to the adsorbed CO; (v) one H on the facet and another H on the edge, both are the first NN to the adsorbed CO; (vi) two H atoms on the facets as the first NNs to the adsorbed CO. The presence of H on the overpotential is summarized in Fig. 5. We find that the effect of H is highly local. Only when H is the nearest neighbour to CO on the edge, would the overpotential increase considerably. All other H adsorption sites have a negligible effect on the overpotential, even when there are two NN H atoms on the facets, as shown in Fig. 5(f). Therefore, we conclude that the presence of H on the NWs would not affect CO_2_ reduction significantly under normal conditions.

### Effects of strain

**Fig. 6:**
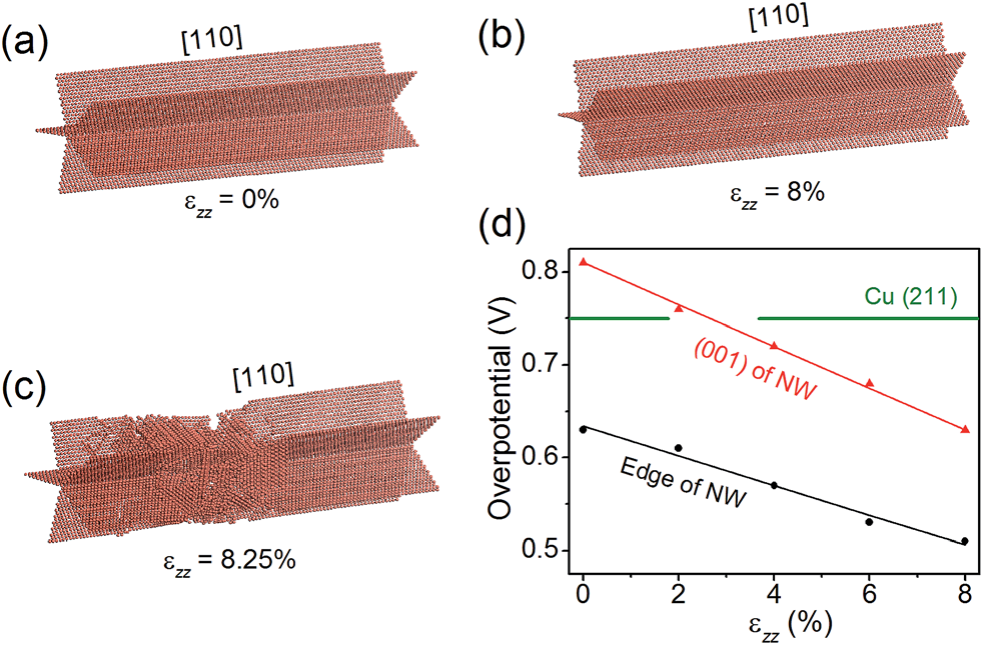
(a–c) The atomic structure of the penta-twinned Cu NW under *ε*_*zz*_ = 0%, 8% and 8.25%. Only the twin boundaries and other lattice defects are shown. (d) Overpotential on the NW edge and (001) facet as functions of uniaxial tension *ε*_*zz*_. The green horizontal line indicates the overpotential on the Cu (211) surface.

Nano-twinned Cu is known to possess a rare combination of ultrahigh mechanical strength and high electrical conductance.^34^ It is also known that elastic strain can modulate adsorption energies, and thus can be used as a “knob” to control the catalytic activity if the strain is large enough. Recent molecular dynamics (MD) simulations have shown that the penta-twinned Ag NWs can withstand an elastic tensile strain of ∼5%, which is orders of magnitude higher than the corresponding value of the bulk material.^35^ Here we perform similar MD simulations to estimate the maximal elastic strain in a penta-twinned Cu NW with *d* = 8 nm. In the simulations, a uniaxial tensile strain *ε*_*zz*_ is applied along the wire direction [110] with a strain rate of 10^7^ s^−1^. Fig. 6(a–c) display the atomic structure under the uniaxial tension of *ε*_*zz*_ = 0%, 8% and 8.25%, respectively. It is found that the Cu NW can maintain its mechanical stability until *ε*_*zz*_ = 8.25%, when necking appears with the formation of stacking faults and other defects. As shown in Fig. 6(c), the stacking faults expand along the {211} planes and intersect with the twin boundaries. Thus it is the combination of the nanoscale dimension and the presence of twin boundaries that gives rise to the ultrahigh mechanical strength of the penta-twinned NWs. The yield strain of the Cu NWs is estimated as 8%, orders of magnitude higher than that of the coarse-grained Cu. Prior to the yield strain, the NW remains elastic due to the absence of lattice defects, and thus can be loaded and unloaded repeatedly for catalysis. Next we calculate the overpotential for CH_4_ production on the penta-twinned Cu NW as a function of tensile strain.

The free energy diagrams of the NW are calculated in the same way as the strain-free case, except now the NW is subject to a tensile strain of *ε*_*zz*_ = 2%, 4%, 6% and 8%. For each strain, we find that CO* → CHO* remains the overpotential-determining step. The overpotential *U*_op_ can thus be expressed as a linear function of *ε*_*zz*_, as displayed in Fig. 6(d):

3 *U*_op_(edge) = −1.60*ε*_*zz*_ + 0.634;

*U*_op_(001) = −2.25*ε*_*zz*_ + 0.810.

Remarkably, we find that the overpotential on the NW edge can be lowered to 0.51 V under 8% tensile strain, which is a 32% reduction relative to the bulk value. The overpotential on the NW (001) facet decreases even faster, and under 2.7% tension, it drops below that on the (211) surface. Hence, with a moderate tensile strain of 2.7%, the entire surface of the NW becomes more active than the bulk Cu. Moreover, the tensile strain is found to suppress the competing HER. For example, under 8% tensile strain, the HER overpotential on the NW edge increases 60% from its strain-free value. Therefore, applying tensile strains is an effective means to significantly enhance the CO_2_ electroreduction activity on the Cu NWs.

### Kinetics of CH_4_ production

We have also calculated the free energy diagrams for the production of ethylene on the NW edge, and the results are detailed in the ESI.† Two reaction pathways have been examined and the overpotential for C_2_H_4_ synthesis is determined by the dimerization of CH_2_, *i.e.*, CH_2_* + CH_2_* → C_2_H_4_*. The overpotential is estimated as 0.82 V, 30% higher than that for CH_4_ production.

**Fig. 7:**
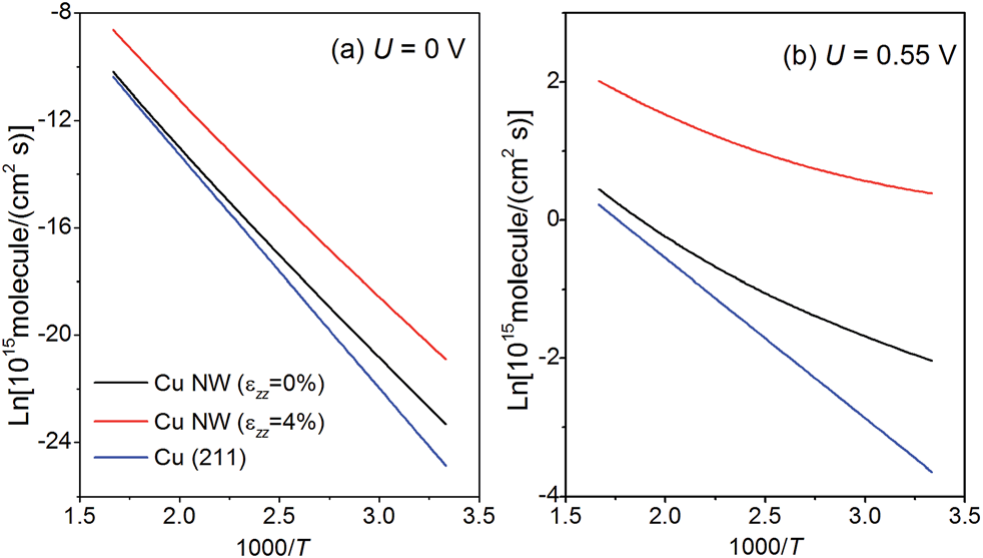
Arrhenius plot for CH_4_ production under a bias potential of (a) *U* = 0 V and (b) *U* = 0.55 V on both the penta-twinned Cu NW (*d* = 24 nm) and Cu (211) surface. The temperature *T* varies from 300 K and 600 K.

In the following, we discuss the kinetics of CO_2_ electroreduction based on a model proposed by Nørskov *et al.*^31^ In this model, the rate of CH_4_ production is assumed to depend only on the activation barrier of the rate-limiting step, CO* → CHO*, and once the intermediate CHO is formed, it must transform to CH_4_. Based on this model, we can express the rate of CH_4_ formation on the NWs as:

4 *r*_NW_(*U*) = *ρ*(edge)*k*_0_e^−*e*[*U*_op_(edge) − *U*]/*k*_B_*T*^ + *ρ*(001)*k*_0_e^−*e*[*U*_op_(001) − *U*]/*k*_B_*T*^,

in which the contributions from both the edges and (001) facets are taken into account. *k*_0_ is the proton transfer rate onto the metal surface, taking a value of 200 s^−1^ site^−1^, as estimated by Nørskov *et al.*^52^ The strain-dependent overpotentials are given in eqn (3). The area density of the adsorption sites on the NW edge, *ρ*(edge), and on the (001) facet, *ρ*(001), can be expressed as:5
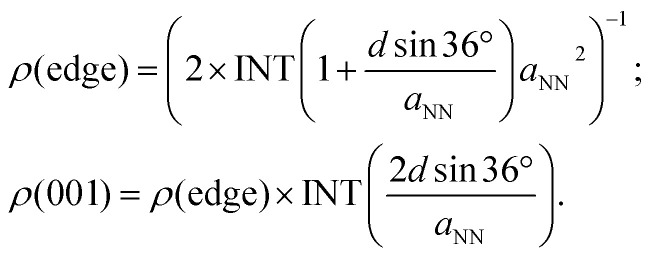
In eqn (5), INT is the downward rounding function and the factor of 2 in *ρ*(edge) accounts for the fact that each CHO would occupy two lattice sites. When *d* = 24 nm, as reported in experiments,^32^*ρ* (edge) and *ρ*(001) are 1.4 × 10^13^ cm^−2^ and 7.5 × 10^14^ cm^−2^, respectively. In Fig. 7, we present the Arrhenius plot for CH_4_ production with *d* = 24 nm. The logarithm of the rate with *ε*_*zz*_ = 0% and 4% under a bias potential *U* = 0 V and −0.55 V *vs.* RHE is displayed in the figure. The same set of plots on the bulk (211) surface is also shown for a comparison with the area density of the lattice sites, given by *ρ*(211) = 3 × 10^14^ cm^−2^. As shown in Fig. 7(a), at *T* = 300 K, *r*_NW_(0 V) is 5 times higher than the rate on the (211) surface; with *ε*_*zz*_ = 4%, *r*_NW_(0 V) can be further enhanced to 56 times the rate on the (211) surface at 300 K. We find that in the practical temperature range between 300 K and 600 K, the production rate of CH_4_ on the Cu NW is always higher than that on the (211) surface, even without straining. We can further compute the current density of CH_4_ production as *i*(*U*) = 8*e* × *r*_NW_(*U*), because 8 electrons are transferred in the process. Under a bias potential of 0.55 V at 300 K, the current density on the Cu NW can reach 2 mA cm^−2^ with *ε*_*zz*_ = 4% (see Fig. 7(b)). In contrast, a bias potential of 1.0 V has to be supplied to reach the same current density on the conventional Cu electrode.^11^

When the penta-twinned NW is unloaded at room temperature, only the edges are activated as they contribute to 95% of the total CH_4_ production rate. However, under a tension *ε*_*zz*_ = 4%, the (001) facets are also activated and contribute to 15% of the total production rate at room temperature. As the temperature ramps up, the area density of the lattice sites plays an increasingly important role in the production of CH_4_. As a result, the contribution from the (001) facets becomes greater, and at *T* > 440 K, their contribution would dominate that from the edges.

### Applying strain *via* graphene

**Fig. 8:**
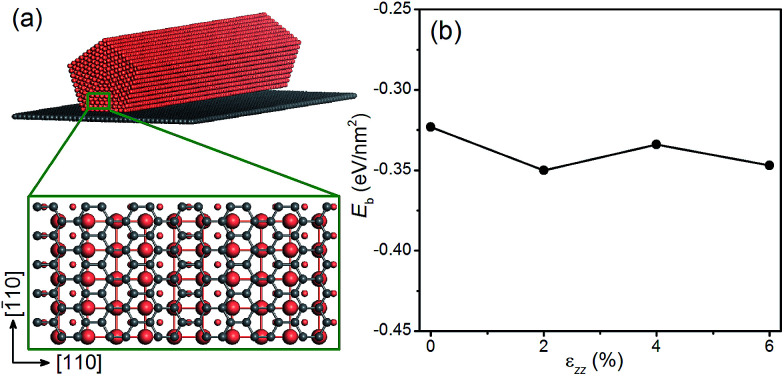
(a) Schematic of a penta-twinned Cu NW on a graphene substrate. The grey and orange spheres represent C and Cu atoms, respectively. The blown-up view of the DFT supercell is shown in the lower panel. (b) Binding energy of the Cu NW on the graphene as a function of the uniaxial tension.

Next we examine the possibility of using graphene as a substrate to stretch the Cu NWs. Since graphene is known to withstand large elastic strains,^55,56^ we propose to deposit Cu NWs on graphene and stretch the graphene appropriately so that the Cu NWs experience the desired tensile strain. This is a plausible proposal since the Cu substrate has been used to grow graphene with a large area and high quality,^57^ and there is experimental evidence for stretching graphene mechanically.^58^ To explore the theoretical feasibility of stretching graphene with deposited Cu NWs, we calculate the binding energy of a penta-twinned Cu NW on graphene as a function of uniaxial tensile strain applied to the graphene. The atomic structure of the combined system is shown in Fig. 8(a). The graphene is parallel to the NW (001) surface. The computational supercell contains 94 atoms, as displayed in Fig. 8(a). The binding energy *E*_b_ as a function of the strain *ε*_*zz*_ along the [110] direction is shown in Fig. 8(d). The negative values of the binding energy indicate the bonding between the two components. In the entire range of the strain, *E*_b_ is found to be negative without a significant change to its value. Hence the ionic bonding between the Cu NW and the graphene appears rather strong and remains so under the tension. Thus it is feasible to apply the desired strain to Cu NWs by stretching the graphene substrate. In the same vein, one can imagine that other materials with a high yield strength may also be used as the substrate.

## Conclusions

In summary, we predict that penta-twinned Cu NWs could exhibit outstanding catalytic activities for CO_2_ electroreduction. The edges of the NWs are predicted to have an overpotential for CH_4_ production that is 16% lower than the most active surface of the conventional Cu electrode. We show that the penta-twinned Cu NWs can withstand extremely high elastic strains, which could be used to further enhance their catalytic activities. An 8% uniaxial tension would lower the overpotential at the NW edges by more than 30%. Moreover, the catalytic activity on the (001) facets of the NWs is also enhanced and can exceed that on the bulk (211) surface with 2.7% tension. On the other hand, the competing side reaction, the HER, on the Cu NWs can be suppressed by the tension strains. The presence of H on the NW surface is found to have a minor effect on CO_2_ electroreduction. We propose that graphene may be used as a substrate to supply desired strains to the overlaying Cu NWs. In conclusion, this work may inspire future research to explore other nano-twinned materials for catalysis.

## Supplementary Material

SC-006-C5SC02667A-s001
