# Supplementary material for: Overpotential for CO2 electroreduction lowered on strained penta-twinned Cu nanowires
Source: Chem Sci. 2015 Aug 19;6(12):6829–35. doi: 10.1039/c5sc02667a (PMC5508656; doi:10.1039/c5sc02667a)
Supplement: SC-006-C5SC02667A-s001 [file SC-006-C5SC02667A-s001.pdf]

Electronic Supplementary Material (ESI) for Chemical Science.
This journal is © The Royal Society of Chemistry 2015

Supplymentary information for

"Overpotential for CO_2 ElectroreductionLowered on Strained Penta-Twinned Cu Nanowires"

Zhengzheng Chen, Xu Zhang, Gang Lu[*]

Department of Physics and Astronomy, California State University, Northridge, CA 91330, USA

Computational Details

Molecular Dynamics (MD) Simulations: The MD simulations are performed using LAMMPS package [1] with the embedded-atom-method (EAM) potential [2] for Cu. The computational unit cell contains ~40000 atoms and the calculated lattice constant of Cu is 3.615 Å. The Cu nanowire is first equilibrated at 100 K for 100 fs, and then relaxedin the *NPT* ensemble at 50 K for 100 ps. The nanowire is then loaded along the long(z) axis at a constant strain rate of 1×10^7/s. Thetemperature is kept at 50 K by the Nosé−Hoover thermostatduring the simulation.

First-principles Density Functional Theory (DFT) Simulations: The computational unit cell is consisted of 150 atoms as shown in Fig. 1c. The DFT calculations are performed using the Vienna ab initio simulation package[3-5]. The revised Perdew-Burke-Ernzerhof (RPBE) exchange-correlation functional [6] and the projected-augmented-wave pseudopotential [7] are used in the calculations. The Brillouin-zone integration is performed with a 2×6×1 *k*-mesh according to the Monkhorst-Pack scheme [8]. The energy cutoff is 400 eVand a Fermi-Dirac smearing width of 0.02 eV is employed. The optimized atomic geometries are achieved when forces on all the unconstrained atoms are

smaller than 0.03 eV/Å. The stabilizing effect of surrounding water layer was also considered with relevant values taken from reference [9]: Adsorbates CO* and CHO* are stabilized by 0.1 eV; COOH* is stabilized by 0.25 eV and OH* is stabilized by 0.5 eV. Therefore, $\Delta G(CO_2(g) \rightarrow COOH^*)$ decreases by 0.25 eV,$\Delta G(COOH^* \rightarrow CO^*)$ increases by 0.15 eV, and$\Delta G(CO^* \rightarrow CHO^*)$ does not change. For the last two steps, $\Delta G(O^* \rightarrow OH^*)$ decreases by 0.5 eV, and $\Delta G(OH^* \rightarrow H_2O\ (g))$ increases by 0.5 eV.

Computational Hydrogen Electrode Model

Following the work of Peterson et al. [9], we calculate the free energy diagrams for CO_2 reduction using so-called “Computational Hydrogen Electrode” (CHE) model [10], in which the reaction

$H^+ + e^- \leftrightarrow H_2(g)/2$

is defined to be in an equilibrium with gaseous H_2 at pressure of 1 atm. at a zero bias voltage, any pH values and temperature.Therefore, in the context of the CHE model,we have the following relation in chemical potential μ of various species at the zero bias voltage

$\mu(H^+) + \mu(e^-) = \mu(H_2(g))/2.$

With this relation, instead of computing the chemical potential of the proton and electron pair, whichis very difficult, we focus on the chemical potential of gaseous H_2. $\mu(H_2(g))$ is taken as -6.884 eV throughout the current work.

The free energy diagram for $CO_2 \rightarrow CH_4$ can be calculated based on CHE model. The correction terms, including zero-point energy, entropy and heat capacity have been included in the calculations:

$$G = E + E_{\mathrm{ZPE}} - TS + \int C_P dT .$$

All correction terms are calculated based on the molecular vibration analysis [11]. Listed in Table S1 are the correction terms of CO* and CHO* under ε_{zz} = 0% and 8%, respectively.

Table S1 Correction terms on the free energy G for intermediates CO* and CHO*

ε_{zz} (%)	Intermediate	E_{ZPE} (meV)	$-TS$ (meV)	$\int C_P dT$ (meV)
0	CO*	181	-140	74
	CHO*	460	-139	73
8	CO*	169	-106	58
	CHO*	465	-123	68

Production of C_2H_4

As reported by previous experiments, C_2H_4 is a common product of CO_2 reduction on Cu electrode. C_2H_4 is also a representative of higher order hydrocarbon fuels containing C-C bonds. Based on previous experimental and theoretical studies [12-14], two relevant pathways are explored in the present work.

The first pathway is shown as Scheme 1:

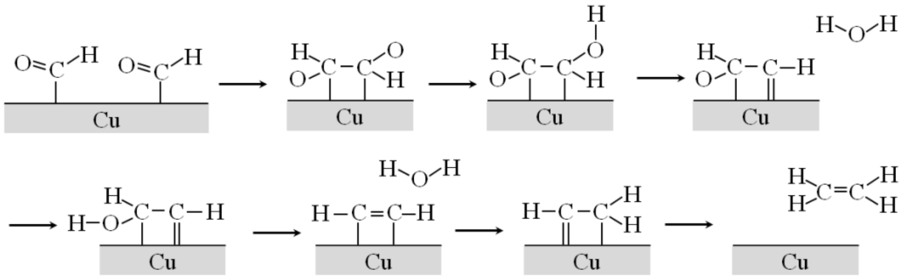

Scheme 1. Proposed first reaction pathway for the production of C_2H_4.

In Scheme 1, the C-C bond is formed by the dimerization of the intermediate CHO*. Therefore the migration of the CHO on the surface is an important process and should be studied here. The migration pathway of the CHO on the NW edge is shown in Figure S1(a). CHO takes a rotation-hopping-rotation process to achieve the highest mobility. First, the bidentate CHO rotates 90° to form a monodentate configuration in which the CHO is perpendicular to the edge. The barrier of the rotation is 0.32 eV. Next, the

monodentate CHO hops on the edge with a barrier of 0.12 eV. Finally, the meta-stable monodentate CHO overcomes a 0.15 eV barrier to rotate back to the stable bidentate configuration at an adjacent position on the edge. We also calculate the migrating barrier of the CHO* without rotation. The barrier is 0.61 eV, almost doubling the rotation energy barrier. In comparison, the migrating barrier of CHO on the (001) facet is 1.1 eV. Therefore in the following we only focus the production of C_2H_4 on the edge of the NW.

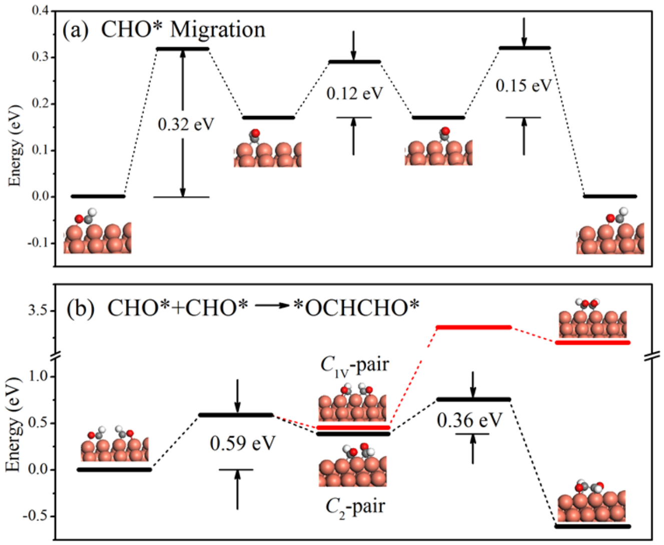

Figure S1. (a) The migration energy barriers of CHO on the edge of the NW. (b) The free energy diagram for the formation of C-C bond. The black and red horizontal lines represent the formation of C-C bond via the C_2-pair and C_{1v}-pair, respectively.

We next explore the C-C bond formation. As shown in Figure S1(b), when two bidentate CHO become the nearest neighbors, they will rotate and form two intermediates after overcoming a 0.59 eV migration barrier. One intermediate is labeled as "C_2-pair" in which the two CHO molecules form C_2 symmetry, and the two O atoms are well separated. Another intermediate is labeled as "C_{1v}-pair" in which two CHO molecules

have the reflection symmetry. C_2-pair is easier to form C-C bond and the energy barrier is 0.39 eV and the formed *OCHCHO* is 1.01 eV lower than the C_2-pair. In contrast, it is energetically very costly for the C_{1v}-pair to form C-C bond. As shown in Figure S1(b), the barrier of the C-C bond formation is 2.9 eV for the C_{1v}-pair. As a result, the rate of C_2H_4 production in this pathway is slow because only half of the CHO-CHO dimers are active to produce C_2H_4.

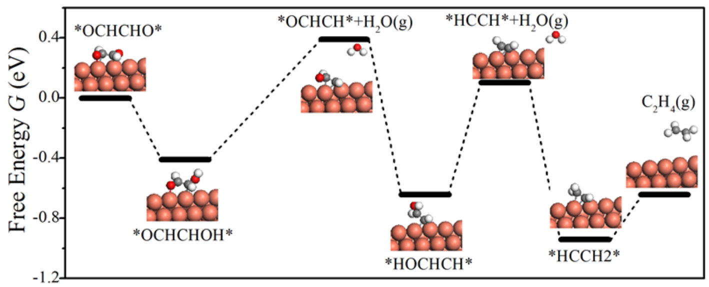

Figure S2. The free energy diagram of the C_2H_4 production.

We next examine the protonation of the *OCHCHO* from the C_2-pair. The free energy diagram is shown in Figure S2. The highest energy barrier is 0.9 eV. Correspondingly, U_{op} of C_2H_4 production is determined as 0.9 V, 43% higher than that of CH_4 production.

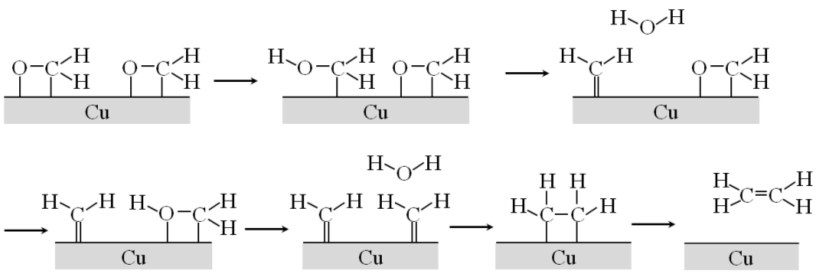

Scheme 2. Proposed second reaction pathway for the production of C_2H_4. CH_2* is produced from the protonation of CH_2O*. The dimerization of the CH_2* leads the production of C_2H_4.

The second pathway is pointed out by a recent study [15], and is shown as Scheme 2. Similar to the first pathway, we have only focused on the edge of the NW. Figure S3 depicts the free energy diagram of the second pathway. The overpotential U_{op} along this pathway is determined by the protonation of CH_2OH^* i.e., $CH_2OH^* \rightarrow CH_2^*+H_2O$ (g), which is 0.81 V, 10% lower than that in the first pathway, but is still 28 % higher than the U_{op} of CH_4 production. On the other hand, C_2H_4 production in the second pathway may be inefficient at lower and medium temperatures since the migration barriers are relatively high. As shown in Figure S3(b), CH_2^* does not have a high mobility along the edge since the migration barrier is 0.82 eV. Desorption of C_2H_4 is another rate-limiting step as it requires an energy barrier of 0.74 eV.

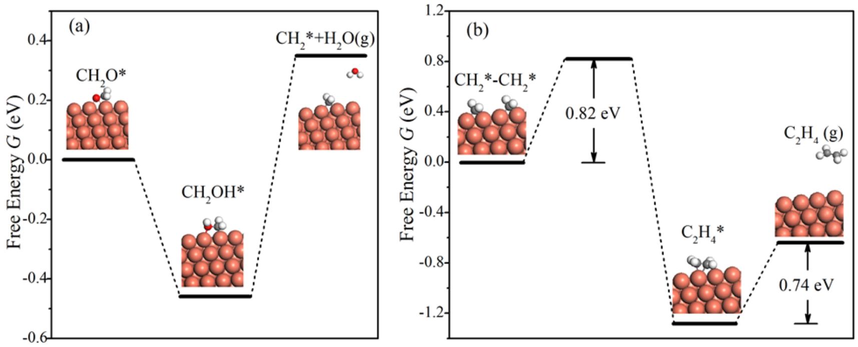

Figure S3. The free energy diagram of the second pathway of the C_2H_4 production.

Coordinates in DFT Calculations

1. Cu NW:

Supercell

33.5491	0.0000	0.0000
0.0000	7.6686	0.0000
0.0000	0.0000	22.3415

Fractional coordinates:

0.186810	0.832430	0.000000
0.186820	0.499480	0.000004
0.186830	0.166520	0.000009
0.687970	0.166600	0.000022
0.687960	0.499540	0.000027
0.687950	0.832500	0.000027
0.937410	0.000117	0.026690
0.937400	0.333070	0.026700
0.937390	0.666030	0.026710
0.124280	0.665950	0.032130
0.124290	0.332990	0.032140
0.124300	0.000026	0.032140
0.750510	0.000117	0.032160
0.750500	0.333090	0.032160
0.750490	0.666040	0.032160
0.061960	0.832420	0.063650
0.061970	0.499460	0.063660
0.061980	0.166510	0.063660
0.812810	0.166600	0.063680
0.812810	0.499560	0.063690
0.812800	0.832510	0.063690
0.249400	0.832430	0.066200
0.249410	0.499480	0.066200
0.249430	0.166520	0.066210
0.625360	0.499540	0.066220
0.625370	0.166590	0.066220
0.625350	0.832500	0.066220
0.000000	0.665940	0.094230
0.000009	0.332980	0.094240
0.000018	0.000026	0.094240
0.874790	0.000130	0.094260
0.874780	0.333090	0.094260
0.874770	0.666040	0.094270
0.186870	0.665950	0.098660
0.186880	0.332980	0.098660
0.186890	0.000026	0.098670
0.687920	0.000130	0.098680
0.687910	0.333090	0.098680
0.687900	0.666040	0.098680
0.124580	0.832420	0.130540
0.124590	0.499460	0.130550
0.124600	0.166500	0.130550
0.750200	0.166620	0.130570
0.750190	0.499570	0.130570
0.750180	0.832520	0.130580
0.312040	0.832430	0.132000
0.312050	0.499480	0.132000
0.312060	0.166520	0.132000
0.562740	0.166590	0.132010
0.562720	0.832500	0.132010
0.562730	0.499540	0.132010
0.062660	0.665940	0.161470
0.062670	0.332970	0.161480
0.062680	0.000013	0.161480
0.812130	0.000143	0.161500
0.812120	0.333100	0.161500

0.812110	0.666050	0.161510
0.249480	0.665950	0.164760
0.249500	0.000026	0.164760
0.249490	0.332990	0.164760
0.625300	0.000117	0.164770
0.625290	0.333090	0.164770
0.625280	0.666040	0.164770
0.437380	0.665990	0.165000
0.437400	0.000078	0.165000
0.437390	0.333030	0.165000
0.187200	0.832420	0.197030
0.187210	0.499450	0.197030
0.187220	0.166500	0.197040
0.687570	0.166620	0.197050
0.687560	0.499570	0.197060
0.687550	0.832520	0.197060
0.374710	0.832450	0.197440
0.374730	0.166540	0.197450
0.374720	0.499490	0.197450
0.500060	0.499530	0.197450
0.500050	0.832490	0.197450
0.500070	0.166580	0.197450
0.125340	0.665920	0.228360
0.125350	0.332970	0.228360
0.125360	0.000013	0.228360
0.749450	0.000143	0.228380
0.749440	0.333100	0.228380
0.749430	0.666050	0.228390
0.312100	0.665950	0.230390
0.312120	0.000039	0.230390
0.312110	0.332990	0.230390
0.562690	0.000117	0.230400
0.562670	0.333070	0.230400
0.562660	0.666030	0.230400
0.437400	0.166550	0.262830
0.437390	0.499520	0.262830
0.437370	0.832470	0.262830
0.249820	0.832420	0.263060
0.249830	0.499460	0.263070
0.249840	0.166500	0.263070
0.624960	0.166620	0.263080
0.624940	0.499570	0.263080
0.624940	0.832520	0.263080
0.188000	0.665920	0.294830
0.188010	0.332970	0.294840
0.188020	0.000000	0.294840
0.686780	0.000143	0.294850
0.686770	0.333100	0.294850
0.686760	0.666050	0.294860
0.374730	0.665980	0.295550
0.374740	0.333010	0.295550
0.374750	0.000052	0.295550
0.500050	0.000104	0.295550
0.500040	0.333060	0.295550
0.500030	0.666020	0.295550
0.312400	0.499460	0.328560

0.312390	0.832430	0.328560
0.312410	0.166510	0.328560
0.562380	0.166600	0.328570
0.562370	0.499560	0.328570
0.562360	0.832510	0.328570
0.437380	0.665990	0.360450
0.437400	0.000078	0.360450
0.437390	0.333030	0.360450
0.250610	0.665940	0.360840
0.250630	0.332970	0.360850
0.250640	0.000013	0.360850
0.624170	0.000143	0.360860
0.624140	0.666050	0.360860
0.624150	0.333100	0.360860
0.373973	0.832741	0.397145
0.373965	0.166650	0.397215
0.373871	0.499705	0.397244
0.500603	0.832791	0.397234
0.500736	0.499710	0.397348
0.500630	0.166686	0.397298
0.311054	0.666419	0.429323
0.311070	0.332681	0.429351
0.311185	0.999595	0.429239
0.563430	0.999674	0.429206
0.563529	0.332785	0.429386
0.563523	0.666500	0.429383
0.437028	0.832953	0.461894
0.437037	0.499748	0.461986
0.437046	0.166600	0.461927
0.373030	0.666739	0.495986
0.373135	0.999793	0.496046
0.373058	0.332833	0.496040
0.500745	0.666704	0.496058
0.500752	0.999804	0.495945
0.500778	0.332934	0.496086
0.436698	0.332661	0.557820
0.436640	0.666738	0.557796
0.436811	0.999794	0.557851

2. Cu NW on a graphene substrate

Supercell

25.56191	0.00000	0.00000
0.00000	2.55619	0.00000
0.00000	0.00000	25.30500

Fractional coordinates

Cu	0.000000	0.000000	0.000000
Cu	0.100000	0.000000	0.000000
Cu	0.200000	0.000000	0.000000
Cu	0.300000	0.000000	0.000000
Cu	0.400000	0.000000	0.000000
Cu	0.500000	0.000000	0.000000

Cu	0.600000	0.000000	0.000000
Cu	0.700000	0.000000	0.000000
Cu	0.800000	0.000000	0.000000
Cu	0.900000	0.000000	0.000000
Cu	0.050000	0.500000	0.071428
Cu	0.150000	0.500000	0.071428
Cu	0.250000	0.500000	0.071428
Cu	0.350000	0.500000	0.071428
Cu	0.450000	0.500000	0.071428
Cu	0.550000	0.500000	0.071428
Cu	0.650000	0.500000	0.071428
Cu	0.750000	0.500000	0.071428
Cu	0.850000	0.500000	0.071428
Cu	0.950000	0.500000	0.071428
Cu	0.000000	0.000000	0.142857
Cu	0.100000	0.000000	0.142857
Cu	0.200000	0.000000	0.142857
Cu	0.300000	0.000000	0.142857
Cu	0.400000	0.000000	0.142857
Cu	0.500000	0.000000	0.142857
Cu	0.600000	0.000000	0.142857
Cu	0.700000	0.000000	0.142857
Cu	0.800000	0.000000	0.142857
Cu	0.900000	0.000000	0.142857
Cu	0.050133	0.500000	0.215063
Cu	0.149912	0.500000	0.215284
Cu	0.249813	0.500000	0.215232
Cu	0.350036	0.500000	0.215170
Cu	0.450115	0.500000	0.215241
Cu	0.550133	0.500000	0.215063
Cu	0.649912	0.500000	0.215284
Cu	0.749813	0.500000	0.215232
Cu	0.850036	0.500000	0.215170
Cu	0.950115	0.500000	0.215241
Cu	0.000318	0.000000	0.287251
Cu	0.099955	0.000000	0.287529
Cu	0.199720	0.000000	0.287476
Cu	0.299808	0.000000	0.287195
Cu	0.400143	0.000000	0.287148
Cu	0.500318	0.000000	0.287251
Cu	0.599955	0.000000	0.287529
Cu	0.699720	0.000000	0.287476
Cu	0.799808	0.000000	0.287195
Cu	0.900143	0.000000	0.287148
Cu	0.050154	0.500000	0.360044
Cu	0.149923	0.500000	0.360147
Cu	0.249744	0.500000	0.359608
Cu	0.350164	0.500000	0.359196
Cu	0.450244	0.500000	0.359451
Cu	0.550154	0.500000	0.360044
Cu	0.649923	0.500000	0.360147
Cu	0.749744	0.500000	0.359608
Cu	0.850164	0.500000	0.359196
Cu	0.950244	0.500000	0.359451
Cu	0.000059	0.000000	0.430095
Cu	0.100192	0.000000	0.430560

Cu	0.200158	0.000000	0.430406
Cu	0.300154	0.000000	0.429370
Cu	0.400078	0.000000	0.429508
Cu	0.500059	0.000000	0.430095
Cu	0.600192	0.000000	0.430560
Cu	0.700158	0.000000	0.430406
Cu	0.800154	0.000000	0.429370
Cu	0.900078	0.000000	0.429508
C	0.999954	0.000000	0.576561
C	0.027389	0.500000	0.576291
C	0.083278	0.500000	0.576097
C	0.110763	0.000000	0.576185
C	0.166640	0.000000	0.576648
C	0.194100	0.500000	0.576912
C	0.249969	0.500000	0.577587
C	0.277426	0.000000	0.577845
C	0.333292	0.000000	0.578058
C	0.360742	0.500000	0.577985
C	0.416626	0.500000	0.577538
C	0.444085	0.000000	0.577262
C	0.499954	0.000000	0.576561
C	0.527389	0.500000	0.576291
C	0.583278	0.500000	0.576097
C	0.610763	0.000000	0.576185
C	0.666640	0.000000	0.576648
C	0.694100	0.500000	0.576912
C	0.749969	0.500000	0.577587
C	0.777426	0.000000	0.577845
C	0.833292	0.000000	0.578058
C	0.860742	0.500000	0.577985
C	0.916626	0.500000	0.577538
C	0.944085	0.000000	0.577262

Reference

1. Plimpton, S. J. *J. Comput. Phys.***1995**, *117*, 1 (http://lammps.sandia.gov).
2. Mishin, Y.; Mehl, M. J.; Papaconstantopoulos, D. A.; Voter, A. F.; Kress, J. D. *Phys. Rev. B.***2001**, *63*, 224106.
3. Kresse, G.; Hafner, J. *Phys. Rev. B.***1993,***47*, 558-561(R).
4. Kresse, G.; Hafner, J. *Phys. Rev. B.***1994,***49*,14251-14269.
5. Kresse, G.; Hafner, J. *Comput. Mater. Sci.***1996,***6*,15-50.
6. Hammer, B.; Hansen, L. B.; Nørskov, J. K. *Phys. Rev. B.***1999,***59*, 7413-7421.
7. Blöchl, P. E. *Phys. Rev. B***1994**, *50*, 17953.
8. Monkhorst, H. J.; Pack, J. D. *Phys. Rev. B.***1976,***13*, 5188-5192.
9. Peterson, A. A.; Abild-Pedersen, F.; Studt, F.; Rossmeisl, J.; Nørskov, J. K. *Energy Environ. Sci.***2010**, *3*, 1311-1315.

10. Nørskov, J. K. Rossmeisl, J.; Logadottir, A.; Lindqvist, L. *J. Phys. Chem. B*. **2004,** *108*, 17886-17892.

11. Cramer, C. J. *Essentials of Computational Chemistry* (Wiley, 2004), 2ndediton.

12. Hori, Y.; Murata, A.; Takahashi, R. *J. Chem. Soc., Faraday Trans. 1***1989**, *85*, 2309-2326.

13. Kuhl, K. P.; Cave, E. R.; Abram, D. N., Jaramillo, T. F. *Energy Environ. Sci.***2012**, *5*, 7050-7059.

14. Durand, W. J.; Peterson, A. A.; Studt, F.; Abild-Pedersen, F.; Nørskov, J. K. *Surf. Sci.***2011**, *605*, 1354-1359.

15. Nie, X. W.; Esopi, M. R.; Janik, M. J., Asthagiri, A. *Angew. Chem. Int. Ed.***2013**, *52*, 2459-2462.
